# Comparing Emotion Recognition Skills among Children with and without Jailed Parents

**DOI:** 10.3389/fpsyg.2016.01095

**Published:** 2016-07-25

**Authors:** Lauren A. Hindt, Laurel Davis, Erin C. Schubert, Julie Poehlmann-Tynan, Rebecca J. Shlafer

**Affiliations:** ^1^Department of Psychology, Loyola University Chicago, ChicagoIL, USA; ^2^Department of Pediatrics, University of Minnesota, MinneapolisMN, USA; ^3^Institute of Child Development, University of Minnesota, MinneapolisMN, USA; ^4^Human Development and Family Studies, School of Human Ecology, University of Wisconsin–Madison, MadisonWI, USA

**Keywords:** parental incarceration, emotion recognition, emotion bias, emotion labeling, jail

## Abstract

Approximately five million children in the United States have experienced a co-resident parent’s incarceration in jail or prison. Parental incarceration is associated with multiple risk factors for maladjustment, which may contribute to the increased likelihood of behavioral problems in this population. Few studies have examined early predictors of maladjustment among children with incarcerated parents, limiting scholars’ understanding about potential points for prevention and intervention. Emotion recognition skills may play a role in the development of maladjustment and may be amenable to intervention. The current study examined whether emotion recognition skills differed between 3- to 8-year-old children with and without jailed parents. We hypothesized that children with jailed parents would have a negative bias in processing emotions and less accuracy compared to children without incarcerated parents. Data were drawn from 128 families, including 75 children (53.3% male, *M* = 5.37 years) with jailed parents and 53 children (39.6% male, *M* = 5.02 years) without jailed parents. Caregivers in both samples provided demographic information. Children performed an emotion recognition task in which they were asked to produce a label for photos expressing six different emotions (i.e., happy, surprised, neutral, sad, angry, and fearful). For scoring, the number of positive and negative labels were totaled; the number of negative labels provided for neutral and positive stimuli were totaled (measuring negative bias/overextension of negative labels); and valence accuracy (i.e., positive, negative, and neutral) and label accuracy were calculated. Results indicated a main effect of parental incarceration on the number of positive labels provided; children with jailed parents presented significantly fewer positive emotions than the comparison group. There was also a main effect of parental incarceration on negative bias (the overextension of negative labels); children with jailed parents had a negative bias compared to children without jailed parents. However, these findings did not hold when controlling for child age, race/ethnicity, receipt of special education services, and caregiver education. The results provide some evidence for the effect of the context of parental incarceration in the development of negative emotion recognition biases. Limitations and implications for future research and interventions are discussed.

## Introduction

The United States (US) has the largest population of incarcerated people in the world (International Centre for Prison Studies, 2015). In 2010, more than half of all incarcerated adults were parents with minor children, resulting in approximately five million children who have experienced a co-resident parent’s incarceration in prison or jail (Murphey and Cooper, 2015). This population of children experiences multiple risk factors for poor developmental outcomes, such as separation from primary caregivers, chaotic living situations, parental substance abuse, stigmatization, and poverty (Eddy and Poehlmann, 2010), and therefore require the attention of researchers and practitioners to effectively promote resilience processes.

Multiple risk factors may drive some of the findings that children with incarcerated parents are at an increased risk for maladjustment and future involvement in the justice system (e.g., Gabel, 1992; Murray and Farrington, 2005; Murray et al., 2012). The associations between parental incarceration and behavioral maladjustment may be partially mediated by general environmental risks (e.g., parent mental health) and exposure to incarceration-specific events, such as witnessing the parent’s arrest (Dallaire et al., 2014). Dallaire et al. (2014) found that children’s experiences related to their mothers’ incarceration (i.e., witnessing arrest, separation from siblings, changing schools) predicted children’s internalizing and externalizing behavior problems. A link between parental incarceration and future behavioral problems has also been noted (Murray and Farrington, 2005). Murray and Farrington (2005) compared rates of criminal behavior among boys in the UK who experienced parental incarceration during their childhood, had an absent parent for other reasons (e.g., hospitalization, death, and family discord), had a parent who was incarcerated prior to the child’s birth, and did not have an absent parent. Parental separation because of incarceration during the first 10 years of the child’s life predicted antisocial temperament and illegal misconduct during adolescence and adulthood (Murray and Farrington, 2005).

Despite several studies linking parental incarceration to adverse outcomes, children with incarcerated parents have also been shown to be resilient in the face of adversity. Extended family support of children with incarcerated parents has been associated with positive developmental and psychological outcomes (Miller, 2007), just as a supportive family environment is associated with resilience in other populations facing adversity (e.g., Masten, 2011; Veronese and Castiglioni, 2013; Veronese et al., 2014). In addition, children with incarcerated parents who have positive relationships with their caregivers and incarcerated parents may have better behavioral outcomes (Dallaire, 2007). For example, perceived caregiver warmth and acceptance have been shown to be associated with fewer behavioral problems among children with incarcerated mothers (Mackintosh et al., 2006). Overall, positive relationships with extended family, caregivers, and incarcerated parents along with continuity of care within these systems have been identified as protective factors for positive adjustment among children with incarcerated parents (Dallaire, 2007).

Adept emotion recognition skills, which develop through interactions with family members (Dunsmore et al., 2009; Davis, 2012), may also serve as a protective factor. Emotion recognition skills have been shown to be related to behavioral regulation and social functioning (Denham, 1986; Denham et al., 1990; Izard et al., 2001; Schultz et al., 2001). The current study sought to examine whether emotion recognition skills differed between children with and without jailed parents.

### Emotion Recognition

Emotion recognition is the perception and interpretation of others’ expressions (Izard et al., 2001; Adolphs, 2002). Emotion recognition skills develop and improve with age, particularly in early childhood (Camras and Allison, 1985), and tend to remain stable over time (Sullivan et al., 2008). The ability to recognize emotions is essential to achieve emotional competence, or one’s ability to utilize emotional skills to interact with the environment and face social problems (Saarni, 1999). Saarni (1999) highlights skills that are essential to behaving appropriately in a social context: recognizing the emotions of oneself and others, grasping the vocabulary of emotions, conveying sympathy and empathy, understanding that one’s internal feelings may not be expressed outwardly, self-regulating to cope with distress, and understanding the role of emotions in relationships (as cited in Davis, 2012). Based on this model, emotion recognition is an essential first step for achieving emotional competence, regulating internal and external behavioral functioning, and yielding positive social outcomes. Consistent with this model, previous research suggests that a child’s ability to appropriately perceive, recognize, and respond to the emotional states of others can predict behavioral and social functioning (Denham et al., 1990).

Children’s emotion recognition abilities are fostered by family and parental interactions (Dunsmore et al., 2009; Davis, 2012). Families’ values and emotional expressiveness influence children’s emotion schemas (Dunsmore et al., 2009). Further, parental expressiveness has been shown to be related to children’s relationships with peers (Cassidy et al., 1992). With a parent’s removal from the home, such as in the case of parental incarceration, a child may have fewer opportunities to be exposed to parental expressiveness and may feel less emotionally supported by the incarcerated parent. Further, the expressiveness of the caregivers and adults left in the household may be changed in many ways (e.g., increased displays of negative affect, as the remaining adults are left to deal with a stressed household). Camras et al. (1988) noted that mothers who had maltreated their children posed expressions that were less recognizable by unrelated observers. Further, mothers’ abilities to pose recognizable expressions significantly correlated with children’s emotion recognition skills (Camras et al., 1988). Based on the evidence suggesting the influential nature of parenting on emotion recognition, the removal of a parent from a child’s life and the experience of concurrent risk factors for poor adjustment (e.g., poverty, housing instability, stigmatization, and dangerous neighborhoods) may disrupt typical emotional development (Pears and Fisher, 2005). On the other hand, adept emotion recognition skills may serve as a protective factor against the removal of a parent and other risk factors for maladjustment faced by children with incarcerated parents.

#### Emotion Recognition Bias

We hypothesize that having a jailed parent could play a role in the development of emotion recognition bias. Emotion processing bias is a component of emotion recognition that can affect behavioral and social outcomes. Valence bias occurs when a child consistently evaluates emotions in an overly positive or negative way (e.g., the tendency to interpret a neutral or positive emotion as negative). Previous studies and the current study have included a neutral face in assessing emotion recognition (Pollak et al., 2000; Mancini et al., 2008; Agnoli et al., 2012) because it does not have a negative or positive direction. Correct labeling of the neutral face indicates exceptional emotion recognition knowledge. Incorrect labeling may provide information regarding a negative or positive emotion processing bias.

Numerous studies with children from at-risk backgrounds have revealed that these children display emotion processing biases. In one study, for example, three- to five-year-olds who had been physically abused demonstrated an emotion response bias for angry labels in comparison to children who had been neglected or were not abused (Pollak et al., 2000). Similarly, children who experienced a traumatic terrorist attack demonstrated an angry and sad response bias in an emotion labeling task, in comparison to children who were not exposed to such environmental risks (Scrimin et al., 2009). These studies indicate that the development of bias in emotion processing is likely affected by one’s environment. Schultz et al. (2000) attempted to isolate the factors that contribute to emotion processing bias by examining emotion recognition among preschoolers from Head Start programs in relation to family insecurity and caregiver depression. Their results indicated that depressed caregivers and an unstable family system predicted an anger labeling bias among the preschoolers (Schultz et al., 2000), which provides support for the claim that parental and familial interactions affect emotional competence and social functioning.

Biases in emotion recognition have been associated with poor behavioral and social outcomes. For example, Schultz et al. (2000) found that, among boys, a bias for angry emotions was positively correlated with aggression and peer rejection. Childhood aggression and peer rejection resulting from emotion processing bias have implications for the perpetuation of hostile attribution bias – a tendency to perceive neutral cues as malicious, yielding aggressive responses and a negative perception of social situations (Dodge and Frame, 1982). This can create a cycle of externalizing behavior, as children’s aggression elicits negative responses from others, thus reinforcing the negative bias.

#### Emotion Recognition Accuracy

In addition to emotion recognition bias, previous research suggests a strong link between emotion recognition accuracy and children’s behavioral functioning. A foundational study by Denham (1986) found that 2- and 3-year-olds with more accurate emotion knowledge were more likely to demonstrate prosocial behavior than children with worse emotion knowledge. A longitudinal study by Izard et al. (2001) provided further support for the relationship between emotion recognition and behavioral functioning – emotion knowledge at age five predicted behavioral functioning and social well-being at age 9 among children from low-income backgrounds.

#### Emotion Recognition among At-risk Populations

Emotion recognition studies with young children from populations facing similar risk factors to children with incarcerated parents suggest that maladaptive contexts of development affect emotion recognition abilities (Schultz et al., 2001; Fries and Pollak, 2004; Sullivan et al., 2008; Fairchild et al., 2009; Scrimin et al., 2009). Studies with children exposed to maltreatment demonstrate related deficits in emotion recognition skills. For example, 4-year-olds with a history of neglect from inner-city New Jersey and Philadelphia performed worse on emotion knowledge tasks compared to children from the same neighborhood who had not experienced neglect (Sullivan et al., 2008). In another example, 4-year-olds adopted from Eastern European orphanages who experienced severe neglect presented deficits in emotion recognition in comparison to children who grew up with their biological parents (Fries and Pollak, 2004). Children with incarcerated parents may share similar sociodemographic risk factors with children who have experienced maltreatment (e.g., trauma and poverty), including separation from a primary caregiver. Pears and Fisher (2005) suggest that removal from a primary caregiver may contribute to the finding that children who were maltreated and in foster care had difficulty understanding emotions compared to a control group. Therefore, it is important to examine emotion recognition skills among children in adverse contexts who experience separation from a parent, like parental incarceration.

### Purpose

The purpose of the current study was to examine emotion recognition skills among children with and without jailed parents. We hypothesized that children with jailed parents would demonstrate a more negative bias in labeling emotions (i.e., report more negative emotions) and less accuracy in labeling emotions compared to children without jailed parents, even controlling for other risk factors (e.g., low caregiver education).

## Materials and Methods

### Participants

Children with jailed parents were recruited as part of a larger study of children’s experiences visiting a parent in jail. The University of Minnesota Twin Cities and University of Wisconsin–Madison Institutional Review Boards and leadership from the partnering jails approved this study. Parents in four jails in two Midwestern states attended information sessions about the study and consented to participate. In total, 284 jailed parents (86.6% fathers) provided consent to participate in the study. Incarcerated parents were given the researchers’ contact information and/or provided their families’ contact information to the researchers. Additionally, some families were recruited in the visiting waiting areas at the jails. Researchers met with families at the jail to describe the study, obtain informed written consent from the caregivers and verbal assent from the children, and administer the study protocol. When families had more than one child in the study age range, a target child was identified by selecting the child in the family who had the most recent birthday. A total of 83 child–caregiver–incarcerated parent triads consented for the current study.

A non-incarcerated comparison group was recruited from the community via a University participant pool. A total of 514 caregivers were contacted by phone and/or e-mail. Most (78.4%) could not be reached, 10.3% declined to participate, and 11.3% agreed to participate. Three families missed their appointments, yielding a comparison group of 55 participants. The study protocol was conducted in a private room at the University of Minnesota Twin Cities.

Of the 138 children who enrolled in the study, 10 were removed from the analyses. Six children with jailed parents and one child in the comparison sample did not complete the task because they were ill, too young to understand the task, or refused to participate. Upon examining the data, two children with jailed parents and one child in the comparison sample labeled each emotion as “good;” these children were deemed as outliers and removed from the sample. In total, 128 participants provided usable data, including 75 children with jailed parents (53.3% male) and 53 without jailed parents (39.6% male). On average, children with jailed parents were 5.37 years (*SD* = 1.69); children in the comparison sample were 5.02 years (*SD* = 1.61).

### Caregiver Surveys

Children’s primary caregivers completed a brief survey, providing information about themselves and the children in their care (e.g., caregiver age, education, income, race and ethnicity, relationship to the child; child age, race and ethnicity). Caregivers reported additional information about the child’s health and development, including special education services.

Nearly half of children with jailed parents were Caucasian (45.9%), followed by African American (32.4%), multiple races (20.3%), and Native American (1.4%). Children without jailed parents were Caucasian (88.7%), multiple races (9.4%), and Asian (1.9%). A minority of children with (13.7%) and without jailed parents (5.7%) identified as Hispanic or Latino. Due to this small sample and little variance in race among children without jailed parents, a dichotomous race/ethnicity variable was created (0 = White/non-Hispanic, 1 = all other races/Hispanic).

Caregiver’s were asked to report their highest level of education. Among caregivers of children with jailed parents, 5.3% had a junior high education, 14.7% some high school, 32% high school, 37.3% some college or specialized training, 6.7% a college degree, and 4.0% a college graduate or professional degree. Few caregivers in the comparison sample had less than a college education; 3.8% had partial college or specialized training. More than half (56.6%) were college graduates, and 39.6% had a graduate or professional degree. Given the limited variability within the comparison group, caregiver education was dichotomized (0 = less than a college degree, 1 = college degree or higher).

Child age, race/ethnicity, receipt of special education services, and caregiver education were included as covariates (see section, Analytic Approach). Income was measured differently across groups and therefore could not be included as a covariate. Caregivers of children with jailed parents were asked to report their *individual* monthly income (*M* = $1,270.50, *SD* = $1,199.28), in contrast, caregivers in the comparison group reported *household* monthly income (*M* = $8,755.33, *SD* = $11,018.28). See **Table 1** for additional demographic information.

**Table 1:** Participant characteristics (*N* = 128).

	Jailed parent sample (*n* = 75)		Comparison sample (*n* = 53)	
	Valid (*n*)	% or *M* (*SD*)	Valid (*n*)	% or *M* (*SD*)
Child receipt of special education services	73	15.1%	53	1.9%
Caregiver gender (female)	71	93.0%	53	92.5%
Caregiver relationship to the child	73		53	
Mother		69.9%		92.5%
Grandmother		17.8%		0%
Grandfather		4.1%		0%
Step-parent		4.1%		0%
Aunt/uncle		2.7%		0%
Father		1.4%		7.5%
Caregiver college graduate	75	10.7%	53	96.2%
Public assistance	75	74.7%	53	1.9%
Caregiver age	73	36.67 (13.09)	53	37.79 (4.63)

### Emotion Recognition Task

A task was adapted from emotion recognition research with children (Denham, 1986; Denham et al., 1990; Schultz et al., 2000, 2001; Miller et al., 2005; Sullivan et al., 2008). Previous research has measured children’s emotion recognition abilities by asking children to label emotions on human faces in photographs. Numerous sets of validated photographs of models expressing common emotions exist. With the target population and time constraints of the current study in mind, a set of validated photos was chosen that possessed an adequate number of models, standardized appearance across models (e.g., clothing, straight-on headshot, etc.), and racial diversity. Further, validated photos were chosen based on inclusion criteria developed through a review of emotion recognition research, which consisted of the five basic emotions (i.e., happy, surprise, sad, angry, and fearful); the neutral expression; and the standardized method of creating expressions, or the Facial Action Code (FAC; Ekman and Friesen, 1976).

The FAC measures facial expressions based on the structure and movements of the face (Ekman and Friesen, 1976). It has demonstrated concurrent validity with other methods of facial measurement (Cohn et al., 1999) and guided the production of photos used by emotion recognition studies among children (Camras and Allison, 1985; Pollak et al., 2000; Fries and Pollak, 2004; Sullivan et al., 2008). Based on its widespread application and previous validation studies, the FAC seemed an appropriate inclusion criterion for the current study’s facial stimuli.

The Radboud Faces Database (RaFD) was chosen, as it met our study criteria and was readily accessible. The RaFD consists of 67 models demonstrating the basic emotions using the FAC (Langner et al., 2010). The RaFD has exceptional validity with an average accuracy score of 82%, 11% higher than other commonly used sets (Langner et al., 2010). Although the RaFD includes Dutch females and males, as well as Moroccan males, there was generally limited racial and ethnic diversity among the models (Langner et al., 2010). However, the RaFD did provide more ethnic diversity than many other available sets, an important selection criterion when considering the diversity of our sample.

A total of 24 photos were chosen from the RaFD (12 females and 12 males). Two females and two males expressed each of the following emotions: happiness, surprise, neutrality, sadness, anger, and fear. None of the models or images were repeated; the stimuli consisted of 24 unique individuals. The photos were printed in black and white and laminated.

Prior to administering the stimuli, each child was asked “How are you feeling today?” Responses were recorded verbatim. Next, the researcher explained to the child that she or he would see photos of people showing different emotions and would be asked to say how the person in each photo felt. The order of the 24 photos was randomized before each administration. The child was shown each photo individually and asked, “How is this person feeling?” Children’s responses were recorded verbatim.

Two coders independently rated each response. Coders demonstrated a high level of agreement, agreeing on 99.51% of decisions. When disagreement occurred, raters discussed the codes until they came to consensus. The coders maintained a list of labels and their respective codes for reference.

Coders rated each response as positive, neutral, or negative. The number of positive and negative labels provided by each child were totaled (up to 24 points total for each valence category). In addition, the number of negative labels provided for neutral and positive stimuli was totaled, yielding a score for negative bias or overextension of negative labels (up to 12 points total).

Children’s responses were also scored one point for each photo when a response with the correct valence was provided (up to 24 points total), yielding valence accuracy score. Happy and surprise required positive descriptors; neutral a neutral descriptor; and sad, angry, and fearful negative descriptors in order to receive points for correct valence accuracy.

Children’s responses were scored one point for correctly identifying the emotion depicted in each of the 24 stimuli, resulting in label accuracy score, also ranging from 0 to 24. Synonyms of the target emotions were accepted and received one point (e.g., “cheerful” was correct for happy and was scored one point).

This coding system yielded five emotion recognition outcomes: the number of positive and negative labels (each ranging from 0 to 24); the number of negative labels provided for neutral and positive stimuli, measuring negative bias/overextension of negative labels (0–12); valence accuracy (0–24); and label accuracy (0–24). Emotion bias or overextension of negative labels (Schultz et al., 2000; Sullivan et al., 2008) and accuracy (Denham, 1986; Denham et al., 1990; Schultz et al., 2001; Miller et al., 2005; Sullivan et al., 2008) have been measured with similar methods in previous literature. Bivariate correlations among key emotion recognition variables with the current sample were in the expected direction and consistent with previous research (Schultz et al., 2000).

### Analytic Approach

To test for group differences in each of the five emotion recognition outcomes (i.e., number of positive and negative labels; negative bias/overextension of negative labels; valence accuracy; label accuracy), we conducted two sets of analyses. First, we examined differences in emotion recognition between children with and without jailed parents using multivariate analysis of variance (MANOVA). Differences between groups were examined with analysis of variance (ANOVA). Second, we tested whether these group differences were maintained with the inclusion of relevant covariates (i.e., child age, race/ethnicity, receipt of special education services, and caregiver’s education) using multivariate analysis of covariance (MANCOVA). Prior to conducting analyses, assumptions for MANOVA and MANCOVA were tested, including basic assumptions (e.g., independence, outliers, linear relationship between independent and dependent variables) along with tests for univariate and multivariate normality, multicollinearity among the dependent variables, and homogeneity of the variance–covariance matrices (Field, 2013; Pepe and Addimando, 2014). Homogeneity of regression slopes is an assumption for MANCOVA, but was not relevant given there were no significant results with the MANCOVA (Field, 2013).

## Results

See **Table 2** for descriptive statistics and bivariate correlations among key variables. The emotion recognition outcomes (with the exception of positive labels provided) had skewness and kurtosis between -2 and 2, indicating normality (Hopkins and Weeks, 1990). The number of positive labels provided had kurtosis of 2.69, which is greater than the typical cutoff (Hopkins and Weeks, 1990). However, MANOVA is robust to violations of normality and if the samples are assumed to be symmetric with the largest variance less than four times the smallest variance, as is the case in the present study, MANOVA is valid (Leech et al., 2005; Howell, 2012). Therefore, we deemed MANOVA and MANCOVA appropriate to use despite possible non-normality.

**Table 2:** Descriptive statistics and bivariate correlations among key variables (*N* = 128).

*Variable*	1	2	3	4	5	6
1. Child age	–					
2. Number of positive emotions labeled	–0.06	–				
3. Number of negative emotions labeled	0.10	–0.54^∗∗∗^	–			
4. Negative bias/overextension	–0.19^∗^	–0.47^∗∗∗^	0.81^∗∗∗^	–		
5. Valence accuracy	0.62^∗∗∗^	0.01	0.10	–0.41^∗∗∗^	–	
6. Label accuracy	0.66^∗∗∗^	0.02	0.03	–0.40^∗∗∗^	0.85^∗∗∗^	–

Range	3–8	0–23	1–24	0–12	9–24	0–24
Mean	5.23	8.64	12.66	2.25	18.23	14.99
*SD*	1.66	3.19	3.32	2.28	3.27	5.01
Skewness	0.28	0.47	–0.09	1.22	–0.38	–0.36
Kurtosis	–1.17	2.69	1.81	1.93	0.16	–0.21

*^∗^*p* < 0.05, two-tailed. ^∗∗∗^*p* < 0.001, two-tailed.*

### Differences in Emotion Recognition between Groups

One-way MANOVA was used to compare children with and without jailed parents on each of the five emotion recognition outcomes. There was a significant effect of having an incarcerated parent on the emotion recognition outcomes (Wilks’ λ = 0.878, *F*(5,122) = 3.40, *p* = 0.007, $\eta_{p}^{2}$ = 0.12). This constitutes a medium to large effect size, indicating a meaningful difference (Cohen, 1988). Separate univariate ANOVAs on the emotion outcome variables revealed a significant main effect of parental incarceration on the number of positive emotion labels provided ($\eta_{p}^{2}$ = 0.04) and negative bias/overextension ($\eta_{p}^{2}$ = 0.04), both of which constituted a small to medium effect size (**Table 3**). Children with jailed parents reported significantly fewer positive emotions (*M* = 8.13, *SD* = 3.36) compared to children without jailed parents (*M* = 9.36, *SD* = 2.82). Additionally, children with jailed parents had more negative bias/overextension (*M* = 2.63, *SD* = 2.45) than children without jailed parents (*M* = 1.72, *SD* = 1.92). There was no main effect of parental incarceration on number of negative emotion labels provided or valence and label accuracy (**Table 3**).

**Table 3:** Descriptive statistics and univariate analysis of variance (ANOVA) drawn from multivariate analysis of variance (MANOVA) in emotion recognition outcomes.

	Jailed parent sample (*n* = 75)	Comparison sample (*n* = 53)	ANOVA			
	Mean (*SD*)	Mean (*SD*)	*df*	*F*	*p*	$\eta_{p}^{2}$
Number of positive emotion labels	8.13 (3.36)	9.36 (2.82)	1,126	4.70	0.032^∗^	0.036
Number of negative emotion labels	13.08 (3.56)	12.06 (2.89)	1,126	2.99	0.086	0.023
Negative bias/overextension	2.63 (2.45)	1.72 (1.92)	1,126	5.09	0.026^∗^	0.039
Valence accuracy	18.25 (3.47)	18.19 (3.00)	1,126	0.01	0.913	0.000
Label accuracy	14.60 (5.46)	15.55 (4.28)	1,126	1.11	0.294	0.009

*^∗^*p* < 0.05.*

### Multivariate Analysis of Covariance

Next, MANCOVA was used to compare children with and without jailed parents on the five emotion recognition outcomes, controlling for child age, race/ethnicity, receipt of special education services, and caregiver education. There were no significant main effects for parental incarceration on any of the emotion recognition outcomes after controlling for these variables (**Table 4**).

**Table 4:** Multivariate analysis of covariance (MANCOVA) in emotion recognition outcomes among children with (*n* = 72) and without (*n* = 53) jailed parents.

	Wilks’ λ	*F*	*df*	$\eta_{p}^{2}$
Intercept	0.17^∗∗∗^	114.51	5,115	0.833
Child age	0.53^∗∗∗^	20.13	5,115	0.467
Race and ethnicity	0.98	0.56	5,115	0.024
Special education	0.97	0.61	5,115	0.026
Caregiver education	0.97	0.83	5,115	0.035
Parental incarceration	0.96	0.94	5,115	0.039

*Data for race/ethnicity were missing for one case; data for receipt of special education services were missing for two cases, hence why the sample size has decreased for the MANCOVA. ^∗∗∗^*p* < 0.001.*

## Discussion

As the number of children with incarcerated parents grows (Schirmer et al., 2009), it is important to examine the developmental processes that may be related to adjustment in the context of incarceration. This study is unique as it examines a developmental variable that is understudied in the literature of children with incarcerated parents: emotion recognition. As hypothesized, children with and without incarcerated parents differed in emotion recognition bias; children with jailed parents demonstrated a more negative emotion labeling bias – or overextension of negative labels – compared to children without jailed parents. In addition, children with jailed parents provided fewer positive emotion labels compared to children without jailed parents. However, these findings did not hold when controlling for child age, race, receipt of special education, and caregiver’s education. Contrary to our hypotheses, children with and without jailed parents did not differ on emotion accuracy. Differences in the MANCOVA were largely due to age. Consistent with previous research (Camras and Allison, 1985), child age was associated with higher valence and label accuracy scores, and lower negative bias/overextension in this sample.

There are several possible explanations why parental incarceration was not significantly associated with emotion recognition skills after controlling for key covariates. First, parental incarceration may not uniquely impact children’s emotional recognition skills. This particular adverse childhood experience may impact children differently than other childhood traumas (e.g., neglect) that have been linked with deficits in emotion recognition skills (Sullivan et al., 2008). Alternatively, these findings may be specific to this sample of children with incarcerated parents, as all of the children in the current study had visited their parents in jail. Children who visit their incarcerated parents may have more opportunities for emotional expression and connection to their incarcerated parents, compared to children who have limited or no contact. We also did not take into account how long the parent had been incarcerated. Perhaps children with incarcerated parents had already adjusted to the separation from the parent, resulting in similar emotion recognition skills to children without incarcerated parents. Finally, the null result may also be due to a lack of statistical power. The sample sizes in the current study were relatively small and the statistical analyses were likely underpowered, particularly with the inclusion of several covariates. These are important possibilities to explore in future research.

Overall, these findings are partially consistent with previous research showing negative emotion processing bias in at-risk populations (Pollak et al., 2000; Schultz et al., 2000; Fries and Pollak, 2004; Sullivan et al., 2008; Scrimin et al., 2009). Prior to controlling for key demographic variables, including risks such as low caregiver education, we found that children with incarcerated parents reported significantly fewer positive emotion labels and had more negative emotion bias/overextension than the comparison group. These findings did not hold, however, after including key covariates, suggesting that it is important to include general risk factors when making group comparisons for children with incarcerated parents. Taken together, our findings suggest that initial observed differences in the number of positive labels provided and negative bias/overextension may be due to differences in the groups’ sociodemographic characteristics, rather than children’s experiences of parental incarceration. These findings provide novel information about emotion recognition among children with incarcerated parents and offer a starting point for other investigators seeking to understand developmental processes among these children. The general context of parental incarceration may play a role in emotion processing bias.

Perhaps children with incarcerated parents have more experiences with negative emotions compared to children without incarcerated parents, given their increased exposure to negative experiences (e.g., separation from parents, feelings of loneliness, changing schools; Eddy and Poehlmann, 2010). Thus, children with incarcerated parents may be more familiar with negative emotions and therefore more likely to provide negative labels in an emotion recognition task. Further, in alignment with the emotional development model (Saarni, 1999) mentioned earlier and previous research (Dunsmore et al., 2009; Davis, 2012), caregivers of children with incarcerated parents are particularly stressed and may express or discuss positive emotions less frequently. Such a notion fits with previous research that showed caregivers’ depressive symptoms and family instability predicted an emotion recognition bias toward anger among children in Head Start (Schultz et al., 2000). Children with incarcerated parents may experience negative or stressful environments before parents’ incarceration and may encounter additional stressors during the incarceration (e.g., Poehlmann-Tynan et al., in press). These experiences likely combine to contribute to children’s understanding and interpretation of emotions. Stressful, hostile, or dangerous home environments may ultimately contribute to children’s negative biases in processing emotions.

Previous research suggests a link between negative emotion biases and aggression (Denham, 1986), as well as peer rejection (Schultz et al., 2000). Interpreting a peers’ neutral or positive emotional expression as negative may lead a child with an emotion recognition bias to act inappropriately in response to peers. Given this relationship and the present findings, negative biases in emotion processing may be one mechanism by which externalizing behaviors develop among children with incarcerated parents; however, more research is needed to test this hypothesis. If this finding is consistent across other samples of children with incarcerated parents, interventions that address emotion recognition and labeling may be a valuable area of inquiry.

### Limitations

Although some findings aligned with past emotion recognition research with other high-risk groups (Denham, 1986; Pollak et al., 2000; Schultz et al., 2000; Scrimin et al., 2009), limitations must be considered. The specific emotion recognition task protocol used in the current study has not been validated, but was derived from tasks outlined in previous emotion recognition literature (Denham, 1986; Denham et al., 1990; Schultz et al., 2000, 2001; Miller et al., 2005; Sullivan et al., 2008). As such, the internal and external validity of the task have not been established; therefore, conclusions made from these results may only be applicable within the constraints of the task. Future research should replicate the methods employed in the current study along with validated measures of emotion recognition to ensure generalizability of these findings.

Another limitation of the emotion recognition task was the lack of racial and ethnic diversity of the models, which could affect children’s ability to relate to the images, particularly in a diverse group of children with incarcerated parents. Lack of racial diversity seems to be a common problem in many of the standard facial expression databases and although we used a database that contained some diverse images, these images were not representative of the sample.

A number of children (10) were removed from analyses due to illness, refusal to participate, and failure to understand the task. Nine of these children were three or four years old. The task in the current study required children to produce labels for emotions, which requires vocabulary and recall skills that very young children may not yet possess (Camras and Allison, 1985). Therefore, the present study may not have accurately depicted the emotion skills of all children, notably the youngest children in the sample. Future emotion recognition research should consider other methods to address this issue (e.g., matching expressions to descriptions of affect-laden situations; Camras and Allison, 1985).

There was also no way to determine whether there were differences between families who declined and those who agreed to participate in the study. As mentioned previously, 78.4% of incarcerated parents’ families could not be reached and 10.3% of those contacted declined to participate. Non-participating families may face more risk factors compared to those in the current study, as they may not be able to visit jail due to transportation issues, poverty, or family discord. On the other hand, these families may experience fewer risk factors (e.g., employment that interferes with visiting hours). Future studies should explore factors that contribute to family members’ likelihood of visiting and how these factors contribute to children’s outcomes.

A significant limitation of the current study was the differences between the groups on demographic characteristics. Ultimately, our comparison group was a convenience sample from a university participant pool, which yielded a more aﬄuent and lower-risk comparison group. We attempted to account for these differences between groups by controlling for child age, race and ethnicity, receipt of special education services, and caregiver’s education. It is likely that the groups differed on other characteristics that were not accounted for in the current study. For example, household income likely varied by groups and has important implications for children’s social and emotional development. Unfortunately, income could not be included as a covariate in the present study because the data were not collected in a way that permitted comparison across groups (i.e., caregivers of children with jailed parents were asked to report their *individual* monthly income and caregivers in the comparison sample reported monthly *household* income). Given the non-equivalence of the groups, the applicability of the findings should be interpreted with caution. Still, this study provides a foundation for future emotion recognition research among children with incarcerated parents.

Future studies examining emotion recognition among children with incarcerated parents should seek comparison groups that have comparable demographic characteristics and risk profiles (e.g., poverty, residential mobility, parental substance abuse, chaos in the home, etc.), but have not experienced a parent’s incarceration. It would also be interesting to compare children who are currently experiencing parental incarceration in prison or jail with other groups, such as children with parents who were arrested, but not incarcerated; children with parents on probation or parole; and children with a history of parental incarceration. Larger sample sizes may also improve the robustness of the current findings. Future research should utilize novel recruitment methods to replicate this study with a larger sample and equivalent comparison groups. Children’s verbal ability or general cognitive skills should also be measured and included in analyses, as they have been found to be related to emotion recognition abilities (Sullivan et al., 2008).

Consistent with national trends in parental incarceration (Glaze and Maruschak, 2010), the majority of the jailed parents in the current sample were fathers. As such, these results may not be generalizable to children with mothers in jail or children who are experiencing a parent’s imprisonment. Mothers are more likely than fathers to have lived with their children prior to arrest (Glaze and Maruschak, 2010) and consequently more likely to have modeled and labeled emotions for children while living with them. Children may experience increased risks and disruption when a mother becomes incarcerated compared to a father (Dallaire, 2007), which may compromise children’s emotion recognition abilities. This is a valuable area for future inquiry.

## Conclusion

Previous research has demonstrated the collateral consequences of parental incarceration for children’s health and development (Eddy and Poehlmann, 2010). However, few studies have examined the potential developmental pathways of those associations. In the current study, we found that children with jailed parents stated less positive emotion labels and presented a negative emotion labeling bias – overextending the use of negative emotion labels – compared to children without jailed parents; this finding did not hold after controlling for key covariates. Researchers should continue examining emotion recognition among children with incarcerated parents and its potential role as a mechanism by which parental incarceration confers risk to children. Understanding the potential mechanisms between parental incarceration and children’s adverse outcomes may provide researchers and clinicians with important first steps for targeting prevention and intervention efforts.

## Author Contributions

All of the authors contributed to the study design along with drafting and revising the manuscript. Specifically, RS and JP-T are the co-principal investigators of the overarching project. LH designed the research question with RS. All of the authors collected the data. LH drafted the paper with the support of the other authors. LD assisted with analyses and writing the methods and results. LD, ES, JP-T, and RS helped organize and revise sections. All authors provided final approval of the manuscript.

## Conflict of Interest Statement

The authors declare that the research was conducted in the absence of any commercial or financial relationships that could be construed as a potential conflict of interest.
